# A Cross-Platform Metabolomics Comparison Identifies Serum Metabolite Signatures of Liver Fibrosis Progression in Chronic Hepatitis C Patients

**DOI:** 10.3389/fmolb.2021.676349

**Published:** 2021-08-03

**Authors:** Meera Shanmuganathan, Mohammad Omair Sarfaraz, Zachary Kroezen, Holly Philbrick, Richel Poon, Andrew Don-Wauchope, Marco Puglia, David Wishart, Philip Britz-McKibbin

**Affiliations:** ^1^ Department of Chemistry and Chemical Biology, McMaster University, Hamilton, ON, Canada; ^2^ Department of Pathology and Molecular Medicine, McMaster University, Hamilton, ON, Canada; ^3^ Department of Medicine, Division of Gastroenterology, McMaster University, Hamilton, ON, Canada; ^4^ Departments of Biological Sciences and Computing Science, University of Alberta, Edmonton, AB, Canada

**Keywords:** metabolomics (OMICS), capillary electrophoresis-mass spectrometry, nuclear magnetic resonance, serum, biomarkers, liver fibrosis, hepatitis C virus infection

## Abstract

Metabolomics offers new insights into disease mechanisms that is enhanced when adopting orthogonal instrumental platforms to expand metabolome coverage, while also reducing false discoveries by independent replication. Herein, we report the first inter-method comparison when using multisegment injection-capillary electrophoresis-mass spectrometry (MSI-CE-MS) and nuclear magnetic resonance (NMR) spectroscopy for characterizing the serum metabolome of patients with liver fibrosis in chronic hepatitis C virus (HCV) infection (*n* = 20) and non-HCV controls (*n* = 14). In this study, 60 and 30 serum metabolites were detected frequently (>75%) with good technical precision (median CV < 10%) from serum filtrate samples (*n* = 34) when using standardized protocols for MSI-CE-MS and NMR, respectively. Also, 20 serum metabolite concentrations were consistently measured by both methods over a 500-fold concentration range with an overall mean bias of 9.5% (*n* = 660). Multivariate and univariate statistical analyses independently confirmed that serum choline and histidine were consistently elevated (*p* < 0.05) in HCV patients with late-stage (F2-F4) as compared to early-stage (F0-F1) liver fibrosis. Overall, the ratio of serum choline to uric acid provided optimal differentiation of liver disease severity (*AUC* = 0.848, *p* = 0.00766) using a receiver operating characteristic curve, which was positively correlated with liver stiffness measurements by ultrasound imaging (*r* = 0.606, *p* = 0.0047). Moreover, serum 5-oxo-proline concentrations were higher in HCV patients as compared to non-HCV controls (*F* = 4.29, *p* = 0.0240) after adjustment for covariates (age, sex, BMI), indicative of elevated oxidative stress from glutathione depletion with the onset and progression of liver fibrosis. Both instrumental techniques enable rapid yet reliable quantification of serum metabolites in large-scale metabolomic studies with good overlap for biomarker replication. Advantages of MSI-CE-MS include greater metabolome coverage, lower operating costs, and smaller sample volume requirements, whereas NMR offers a robust platform supported by automated spectral and data processing software.

## Introduction

High-field nuclear magnetic resonance (NMR) spectroscopy and high-resolution mass spectrometry (MS) are two core instrumental platforms used in discovery-based metabolomics research. Selection of one or more analytical method(s) is dependent on several factors, including infrastructure/operating costs, sample volume/workup requirements, sample throughput, as well as selectivity and sensitivity that impact overall metabolome coverage. In general, NMR offers excellent reproducibility and quantitative performance together with unambiguous metabolite identification, and thus is well suited for longitudinal metabolomic studies, metabolic flux analysis, and non-invasive analysis of tissue specimens (Emwas et al., 2019). However, concentration sensitivity and resolution are limited without isotopic enrichment when using fast spectral acquisition protocols in one-dimensional proton (^1^H)-NMR. These constraints result in quantification of typically a few dozen polar serum metabolites depending on magnet field strength, probe design, and spectral processing strategies (Wishart, 2019; Casadei-Gardini, et al., 2020)*.* NMR metabolite coverage can be further expanded to include various lipoprotein, cholesterol and fatty acid species when ultrafiltration is avoided (Würtz et al., 2017). In contrast, MS-based metabolomic methods are more accessible when using bench-top instrumentation with greater sensitivity and resolution especially when coupled to one or more high efficiency separation techniques (Kuehnbaum and Britz-McKibbin, 2013). For instance, liquid chromatography (LC)-MS using separation mechanisms (e.g., reversed-phase, hydrophilic interaction) provide exceptional metabolome coverage (Rhee et al., 2019) especially when using chemical isotope labeling methods (Han and Li, 2018). Yet, separations in LC-MS are generally constrained by lower throughput and complicated data pre-processing when performing non-targeted metabolite profiling (i.e., time alignment, peak picking), where a major fraction of molecular features comprise compounds with unknown chemical structures (da Silva et al., 2015). Alternatively, multisegment injection-capillary electrophoresis-mass spectrometry (MSI-CE-MS) offers a multiplexed separation platform for metabolomics (Kuehnbaum et al., 2013) with higher sample throughput, improved quality control, and lower sample volume requirements (Nori de Macedo et al., 2017) Furthermore, customized serial injection configurations accelerate biomarker discovery using novel data workflows to encode mass spectral information temporally within a separation (DiBattista et al., 2017) while providing robust inter-batch adjustment in large-scale metabolomic studies (Shanmuganathan et al., 2021). Although there have been several cross-platform metabolomic analysis involving NMR and LC-MS (Nevedomskaya et al., 2011; Psychogios et al., 2011; Bruno et al., 2018; Bhatia et al., 2019), to the best of our knowledge no study to date has explored the potential benefits of combining CE-MS with NMR methodologies in metabolomics (Marshall and Powers, 2017).

Chronic hepatitis C virus (HCV) infection can lead to progressive liver disease with a high risk for death from cirrhosis and hepatocellular carcinoma if not treated early with pangenotypic direct-acting antiviral regimens (Ghany and Morgan. 2020). Most individuals (∼85%) infected with HCV develop chronic infections, which contribute to a high burden of liver-related disease complications and spiraling healthcare costs (Myers et al., 2014). Similar to other forms of chronic liver disease, HCV infections are accompanied by liver fibrosis, a scarring process characterized by thickening of liver tissue and accumulation of extracellular matrix proteins with eventual loss of liver function (Khatun and Ray, 2019). Optimal patient care and treatment decisions are dependent on staging of disease progression, which has relied on a liver biopsy to assess the severity of fibrosis, such as the widely used Meta-analysis of Histological Data in Viral Hepatitis (METAVIR) scoring system (Goodman, 2007). However, liver biopsy is an expensive and invasive procedure with risks for patient bleeding, including other complications. It is also prone to both sampling and inter-subject variability depending on quality of biopsy, and thus has been increasingly supplanted by less invasive methods for liver fibrosis assessment, such as ultrasound imaging and blood-based liver protein panels. As a result, there is growing interest in metabolomics to identify novel biomarkers of hepatic fibrosis that offer greater specificity, sensitivity, and reproducibility, and accessibility in a clinical setting (Chang and Yang, 2019). This is urgently needed to augment diagnostic applications for chronic liver disease differentiation (Soga et al., 2011), monitoring treatment responses to therapy (Meoni et al., 2019), and risk assessment of advanced stages of liver disease (Diren and Idle, 2020), including patients co-infected with HCV/HIV (Naggie et al., 2020).

In this work, metabolomic analyses were performed on serum filtrate samples collected from HCV patients at different stages of liver fibrosis, as well as non-HCV controls when using MSI-CE-MS and ^1^H-NMR. Standardized protocols were used for sample preparation, data acquisition, and data pre-processing allowing for an inter-method comparison of serum metabolites consistently measured by both techniques. Importantly, this cross-platform metabolomics study allowed for independent replication of serum biomarker candidates associated with liver fibrosis progression from chronic HCV infection, which complement tissue histopathology, serum liver protein panels, and ultrasound imaging techniques.

## Results

### Clinical Characteristics of Study Participants

Demographic and clinical characteristics of all study participants, including healthy non-HCV participants (*n* = 14), and patients with chronic HCV infection at different stages of liver fibrosis (*n* = 20) are summarized in Table 1. In this pilot study, most recruited subjects were older, overweight male adults, with non-HCV participants generally being younger (*p* = 0.0318). HCV infected patients were treatment naïve at the time of recruitment with most having a HCV genotype A, as confirmed by positive serum anti-HCV antibodies and HCV RNA test results. Staging of liver fibrosis using the METAVIR scoring system was performed by tissue histopathology which confirmed no fibrosis (F0) in non-HCV controls, whereas minimal scarring/inflammation (early-stage, F0-F1, *n* = 9) or more advanced stages of fibrosis (late-stage, F2-F4, *n* = 11) in HCV patient sub-groups with four individuals having cirrhosis (F4). Similarly, FibroScan test results using transient elastography measurements revealed no differences in liver stiffness between non-HCV and early-stage liver fibrosis HCV patients (FibroScan <7.0 kPa, *p* < 0.05), in contrast to the late-stage fibrosis HCV patients (FibroScan >8.0 kPa, *p* = 1.73 × 10^−4^). As expected, a panel of blood liver proteins and FibroTest scores were elevated in HCV patients as compared to non-HCV controls. However, there were no significant differences in circulating liver protein levels between HCV sub-groups at different stages of liver fibrosis severity (*p* > 0.05).

**TABLE 1:** Study characteristics of treatment-naïve hepatitis C virus (HCV) infected patients (*n* = 20) at different stages of liver fibrosis, and healthy non-HCV infected participants (*n* = 14).

Criteria	Non-HCV (*n* = 14)	HCV early-stage (*n* = 9)	HCV late-stage (*n* = 11)	*p*-value^a^
Age (years)	44 ± 15	57 ± 10	63 ± 11	**0.0318**; 0.216
Sex (male)	10 (77%)	8 (89%)	9 (82%)	—
BMI (kg/m^2^)	24.4 ± 1.3	26.0 ± 5.8	26.5 ± 5.0	0.334; 0.843
FibroScan test (kPa)^b^	4.81 ± 0.55	5.39 ± 1.1	11.2 ± 3.6	0.111; **1.73 × 10^−4^**
FibroTest score^c^	0.22 ± 0.17	0.63 ± 0.23	0.69 ± 0.23	**1.61 × 10^−4^**; 0.592
γ-Glutamyltransferase (U/L)	29 ± 26	132 ± 125	76 ± 77	**8.69 × 10^−3^**; 0.233
Total bilirubin (µM)	11.0 ± 6.3	14.8 ± 7.5	11.8 ± 6.8	0.216; 0.369
Alpha-2-macroglobulin (g/L)	1.82 ± 0.56	3.4 ± 1.2	4.4 ± 1.2	**4.08 × 10^−4^**; 0.0747
Hapatoglobin (g/L)	1.03 ± 0.39	1.09 ± 0.51	1.40 ± 0.75	0.780; 0.337
Apolipoprotein A1 (g/L)	1.36 ± 0.20	1.44 ± 0.11	1.40 ± 0.30	0.311; 0.720
Alanine aminotransferase (U/L)	24.1 ± 8.4	82 ± 55	52 ± 36	**1.16 × 10^−3^**; 0.151
Fibrosis grade/METAVIR score^d^				
F0	14	5	—	—
F1	—	4	—	—
F2	—	—	5	—
F3	—	—	2	—
F4	—	—	4	—
HCV genotype				
1A/1B	—	9/0	9/2	—

a Student’s t-test to assess statistical significance (**p < 0.05**) when comparing healthy non-HCV controls with early-stage HCV, as well as early-stage HCV with late-stage HCV patients, respectively.

b FibroScan test results to assess liver fibrosis based on transient elastography using ultrasound imaging.

c FibroTest uses an algorithm that combines 5 standard serum protein biomarkers, including γ-glutamyltransferase, total bilirubin, alpha-2-macroglobulin, haptoglobin, and apolipoprotein A1.

d METAVIR score to assess the extent of inflammation and fibrosis by histopathological examination of a liver biopsy.

### Serum Metabolome Characterization by Multisegment Injection-Capillary Electrophoresis-Mass Spectrometry

**FIGURE 1:**
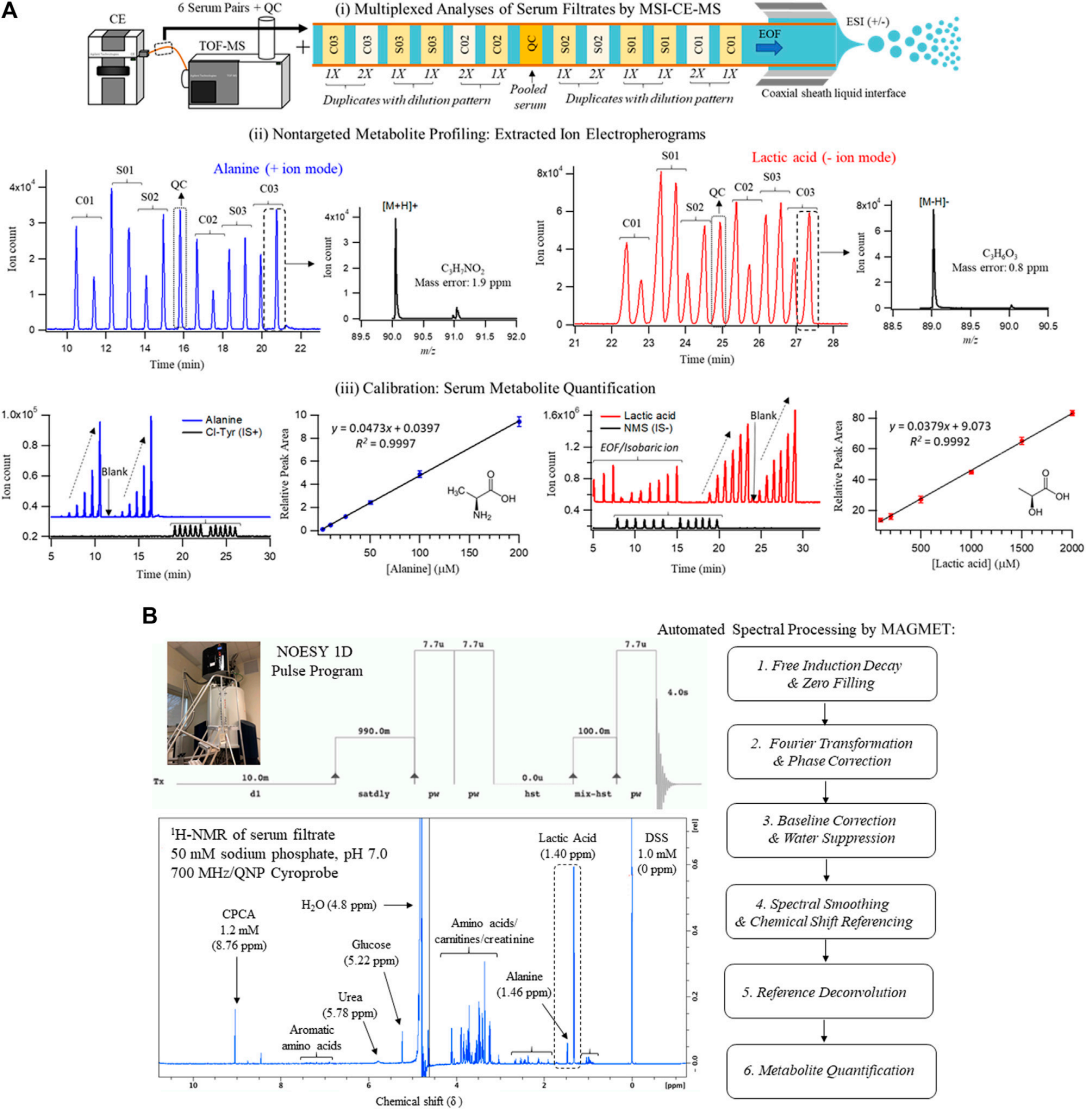
Cross-platform method comparison for characterization of the serum metabolome from HCV patients with liver fibrosis and non-HCV controls when using **(A)** MSI-CE-MS and **(B)** ^1^H-NMR. In both cases, diluted serum filtrate samples were analyzed using standardized protocols, which included 60 and 30 polar/ionic metabolites measured in most samples (>75%) with adequate precision (CV < 35%) by MSI-CE-MS and NMR, respectively. Each sample was analyzed by NMR with signal averaging (120 scans, 12 min/sample) followed by automated spectral processing by MAGMET using a targeted metabolite library, whereas six pairs of serum filtrates together with a pooled QC were analyzed in each MSI-CE-MS run (8 min/sample) using both targeted and nontargeted approaches with metabolite quantification using external calibration curves with internal standards. Serum metabolite concentrations measured consistently by both platforms are illustrated in this case for alanine and lactic acid.

**FIGURE 2:**
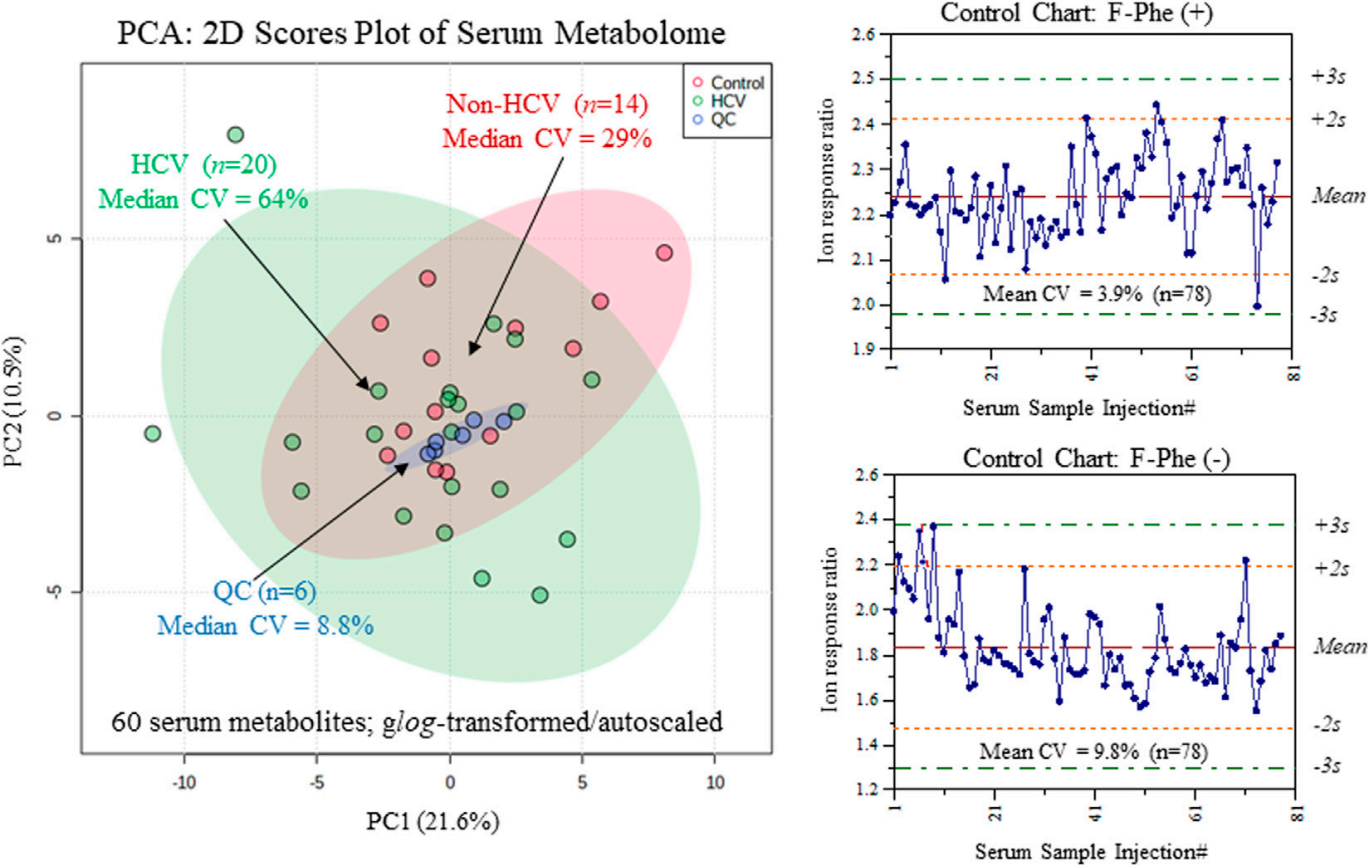
Overview of the serum metabolome and data quality when using MSI-CE-MS as depicted in 2D scores plot using PCA, which compares the technical precision (median CV = 8.8%, *n* = 6) from repeat QC samples relative to the larger biological variance in chronic HCV liver fibrosis patients (median CV = 64%, *n* = 20) and non-HCV controls (median CV = 29%, *n* = 14). Control charts for the recovery standard (3-fluorophenylalanine, F-Phe) confirms acceptable intermediate precision (mean CV = 6.8%, *n* = 156) when analyzing all serum samples in this study by MSI-CE-MS in positive and negative ion modes.

Serum samples were prepared by ultrafiltration to remove protein after dilution with recovery/internal standards prior to MSI-CE-MS analysis, which were analyzed under two configurations for cationic (pH 1.8, positive ion mode) and anionic (pH 8.5, negative ion mode) metabolites with full-scan data acquisition. Sample throughput in MSI-CE-MS is enhanced when using a serial sample injection format comprising 13 serum filtrates analyzed within a single run. In this case, duplicate analysis of each serum filtrate diluted in a distinctive pattern (1:2, 1:1, 2:1) together with a pooled QC sample were acquired in random sequence as shown for alanine and lactic acid in Figure 1A. Data pre-processing in MSI-CE-MS combined both targeted analysis of known serum metabolites, as well as a nontargeted screening strategy to authenticate unknown metabolites from a pooled serum sample as described elsewhere (Shanmuganathan et al., 2021). All serum metabolites were annotated based on their characteristic accurate mass: relative migration time (*m/z*:RMT) under positive (p) or negative (n) ion mode detection after rejecting spurious signals, background ions and dataset redundancy (Saoi et al., 2020). Overall, 55 serum metabolites in this study were identified with high confidence (level 1) after spiking with authentic standards (*i.e.,* co-migration) and having low mass error (<5 ppm). This also allowed for their quantification using an external calibration curve with ion responses normalized to an internal standard (20 μM, 4-chlorotyrosine, Cl-Tyr or naphthalene monosulfonic acid, NMS) over a 100-fold dynamic range with good linearity (*R* ^*2*^ > 0.990). Otherwise, unknown metabolites (5 compounds, level 4) were annotated based on their most likely molecular formula. Overall, 60 polar/ionic metabolites were consistently analyzed (median CV < 35%) in most serum samples (>75%) from non-HCV controls and HCV patients (*n* = 34) by MSI-CE-MS. Figure 2 depicts a 2D scores plot from principal component analysis (PCA) confirming acceptable technical precision achieved for 60 serum metabolites from QC samples (median CV = 8.8%, *n* = 6) relative to the larger biological variance in non-HCV (median CV = 29%, *n* = 14) and HCV patients (median CV = 64%, *n* = 20). Also, control charts for the recovery standard (20 μM, 3-fluorophenylalanine, F-Phe) added to all serum samples and analyzed under both MSI-CE-MS configurations demonstrated good intermediate precision (mean CV = 6.8%, *n* = 156) with few data (∼5%) exceeding warning limits (±2s).

### Targeted Metabolite Profiling of Serum Filtrates by Nuclear Magnetic Resonance

A standardized approach was also used to prepare serum samples (280 μl) after ultrafiltration to remove protein followed by a 1.25-fold dissolution in a buffer system (70 μl, 250 mM phosphate, pH 7.0 with 54% *vol* D_2_O) containing a chemical shift reference that also served as internal standard (1.0 mM 2,2-dimethyl-2-silapentane-5-sulfonate, DSS-d6). ^1^H-NMR spectra (700 MHz) in 5 mm diameter tubes were acquired using a NOESY pulse sequence with water suppression after a manual shimming protocol, which generated an average line width for DSS-d6 of (0.95 ± 0.14) Hz (*n* = 40). Each ^1^H-NMR spectrum required 12 min for 128 scans as shown for a pooled serum filtrate sample from the study cohort in Figure 1B. Most proton resonances for serum metabolites have signals clustered within distinct chemical shift windows (δ ∼ 3.2–4.2 ppm; 1.8–2.7 ppm; 0.8–1.2 ppm) as compared to prominent peaks for lactic acid (methyl proton, δ = 1.40 ppm, doublet), alanine (methyl proton, δ = 1.46 ppm doublet), and *d*-glucose (α-anomeric proton, δ = 5.22 ppm, doublet). Raw FID NMR data was uploaded to a user-friendly webserver, Magnetic Resonance for Metabolomics (MAGMET) using an automated workflow for data pre-processing and spectral deconvolution from a library of 47 serum metabolites (Ravanbakhsh et al., 2015). After spectral processing, serum metabolite concentrations were calculated using a reference standard with known concentration (DSS-d5, 1,000 µM). Technical precision was assessed from the intermittent analysis of an external QC comprised of four amino acid standards (mean CV = 2.5%, *n* = 3), as well as internal QC of pooled serum from cohort (median CV = 9.0%, *n* = 3) which had higher variance (CV > 35%) for certain serum metabolites prone to spectral interferences (*e.g.,* arginine, methionine, leucine, hydroxyvaleric acid). Overall, 30 polar/ionic metabolites were reliably quantified by ^1^H-NMR in most serum samples in this study.

### Serum Metabolite Quantification: Multisegment Injection-Capillary Electrophoresis-Mass Spectrometry vs Nuclear Magnetic Resonance

**FIGURE 3:**
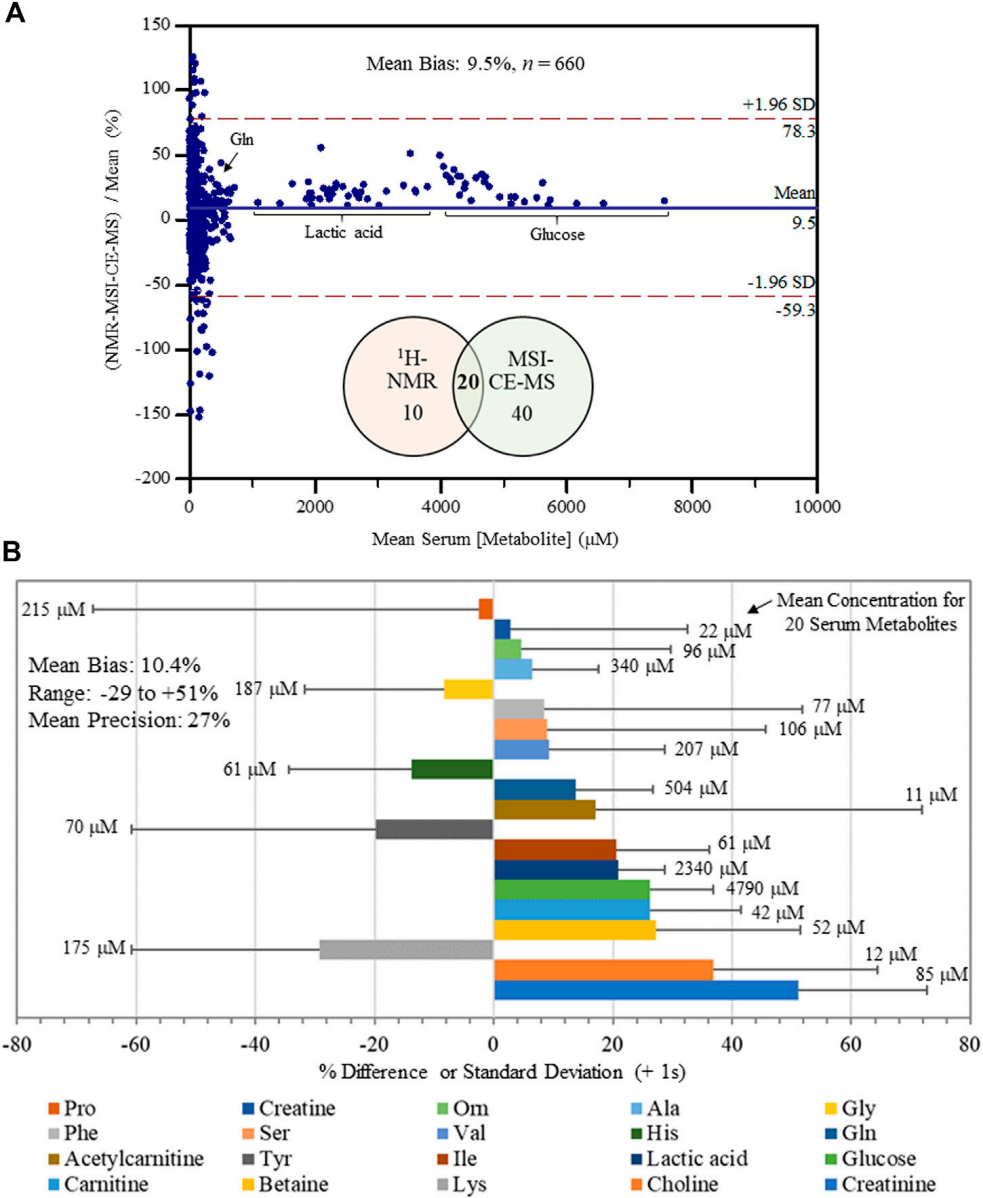
Inter-method comparison for serum metabolite quantification by ^1^H-NMR and MSI-CE-MS platforms in non-HCV and HCV patients (*n* = 34). **(A)** A Bland-Altman %difference plot confirms a normal distribution for 20 serum metabolite concentrations measured independently in 34 serum filtrate samples having a mean bias of 9.5% (*n* = 660) with few metabolites (∼5%) exceeding mutual agreement limits ( ± 2s). **(B)** A summary of the mean concentration (μM) for each serum metabolite consistently measured by both instrumental platforms, as well as a bar graph depicting the mean bias (% difference) together with an error bar indicating the mean precision (+1 s) in reported bias across all samples (*n* = 34). Serum metabolites analyzed in this study were ranked ordered (top to bottom) from lowest to highest bias having an overall bias and precision of 10.4 and 27%, respectively.

An inter-method comparison was next performed for serum metabolites measured with high detection frequency (>75% of all samples analyzed) and adequate technical precision (CV < 35%) by both MSI-CE-MS and NMR platforms. Concentration detection limits for ^1^H-NMR under the acquisition conditions used were about 5 μM, but higher detection thresholds were evident for certain serum metabolites prone to chemical shift spectral overlap. In MSI-CE-MS, concentration sensitivity is metabolite dependent given the disparity in solute ionization efficiency (Chalcraft et al., 2009) with detection limits (S/N ∼ 3) ranging from 0.2 to 0.5 μM when using a conventional coaxial sheath liquid interface with small volumes (∼5 nL) introduced on-capillary. Figure 3A depicts a Bland-Altman %difference plot highlighting a normal data distribution with an overall mean bias of 9.5% (*n* = 660) based on 20 serum metabolite concentrations measured in 34 serum samples by MSI-CE-MS and NMR with few missing data (20 or 2.9% in total). There was a significant overlap in metabolome coverage (∼67%) between both platforms that comprised primarily micromolar levels of polar/ionic metabolites from serum filtrates. Over a 500-fold dynamic range in serum metabolite concentrations was assessed in non-HCV controls and HCV patients (*n* = 34) ranging from *d*-glucose (mean concentration of 4.8 mM) to *O*-acetyl-*l*-carnitine (mean concentration of 11 μM). Bias was evident among a sub-set of samples/serum metabolites (*e.g.,* proline, tyrosine, phenylalanine), yet only a small fraction of total data (∼5.0%) exceeded mutual agreement limits (±2s). Figure 3B illustrates the bias distribution for each of the 20 serum metabolites (mean bias of 10.4% ranging from -29 to +51%) that are depicted as solid bars. Also, the mean precision in measured bias between the two platforms is 27%, which is shown as an error bar (+1s) for each serum metabolite. Overall, serum alanine, glycine, ornithine, valine, histidine, glutamine, isoleucine, lactic acid, glucose, carnitine and betaine had the most consistent measurements across both methods in terms of acceptable bias and variability (<25%) when using only a single internal standard for data normalization. An excel file in the Supplementary Material provides a complete list of serum metabolites and their responses/concentrations measured in non-HCV and HCV patients using MSI-CE-MS and NMR, including calibration curves and figures of merit acquired for 20 serum metabolites used in this inter-method comparison.

### Serum Metabolites Differentiating Liver Fibrosis Progression in Hepatitis C

**FIGURE 4:**
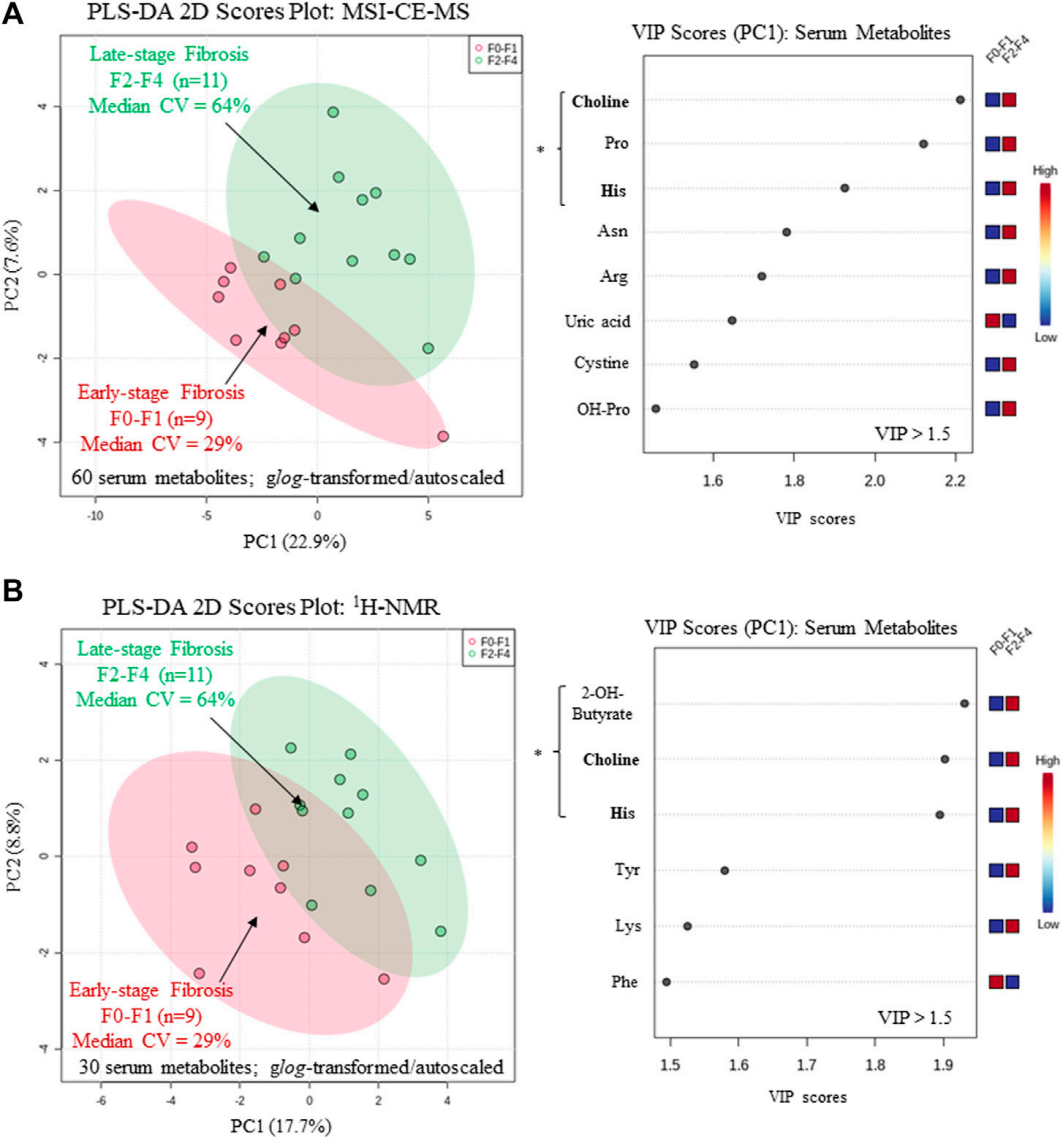
Supervised multivariate data analysis of serum metabolome characterized by **(A)** MSI-CE-MS and **(B)** 1H-NMR when using a partial least-squares-discriminate analysis (PLS-DA) model for differentiation of chronic HCV patients with early (F0-F1) from late-stage (F2-F4) liver fibrosis as determined by histopathology. A variable importance in projection (VIP) scores (VIP >1.5) were used to rank order important serum metabolites associated with liver fibrosis progression highlighting consistent outcomes for choline and histidine (bolded) in both platforms. All serum metabolites were generalized *log*-transformed and autoscaled, where * indicates statistically significant serum metabolites between HCV sub-groups (*p* < 0.05).

A major focus of this pilot study was to identify putative serum biomarkers that differentiate liver fibrosis in treatment naïve HCV patients. Complementary multivariate and univariate statistical analyses were performed on serum metabolome data to identify putative serum biomarkers that may enable less invasive assessment of liver fibrosis. Figure 4 compares two partial least squares-discriminant analysis (PLS-DA) models from MSI-CE-MS and ^1^H-NMR data to rank order significant serum metabolites (VIP >1.5) that differentiate late-stage fibrosis (F2-F4, *n* = 11) from early-stage (F0-F1, *n* = 9) fibrosis in well-matched HCV patients based on their METAVIR scores (Table 1). Overall, serum choline and histidine were among the top ranked metabolites consistently elevated in late-stage as compared to early-stage fibrosis HCV patients by both MSI-CE-MS and NMR. Table 2 confirms that both MSI-CE-MS (choline, proline, histidine) and NMR (2-hydroxybutyric acid, choline, histidine) identified four serum metabolites elevated (mean fold-change, FC > 1.2, *p* < 0.05, effect size >0.90) with more advanced stages of liver fibrosis when using a two-tailed student’s t-test with equal variance. Hydroxybutyric acid isomers were not fully resolved by MSI-CE-MS in this study preventing their accurate quantification, whereas serum proline was not found to be different between HCV sub-groups using NMR, and thus not independently replicated. Several other serum metabolites also had higher serum concentrations with increasing liver fibrosis (*e.g.,* asparagine, arginine, tyrosine, hydroxyproline) in contrast to uric acid and phenylalanine (Figure 4); however, these trends were not statistically significant (*p* > 0.05).

**TABLE 2:** Cross-platform comparison of serum metabolites that differentiate HCV patients with late (F2-F4, *n* = 11) to early-stage (F0-F1, *n* = 9) liver fibrosis.

Serum Metabolite/ID	Mean FC	*p*-value	Effect size^a^
*MSI-CE-MS*	—	—	—
Choline	1.43	0.0312	1.03
Proline	1.41	0.0401	0.99
Histidine	1.19	0.0653	0.90
^*1*^ *H-NMR*	—	—	—
2-Hydroxybutyric acid	2.24	0.0307	1.04
Choline	1.32	0.0447	0.94
Histidine	1.21	0.0456	0.97

a A two-tailed student’s t-test with equal variance of log-transformed serum metabolite concentrations was used to differentiate liver fibrosis progression, where effect size is defined as eta^2^.

**FIGURE 5:**
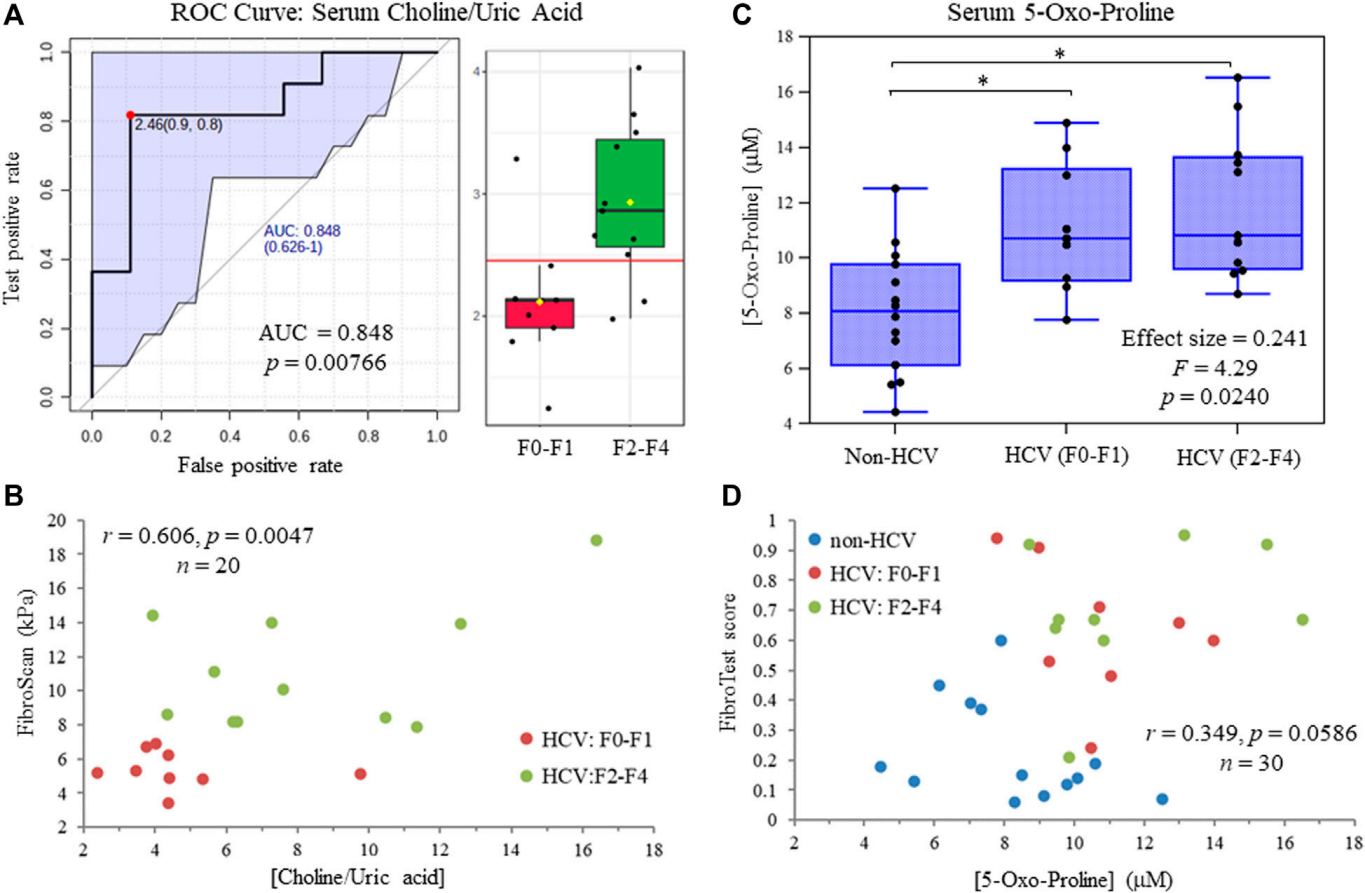
**(A)** Receiver operating characteristic (ROC) curve highlighting that serum choline:uric acid ratio optimally differentiates late (F2-F4) from early (F0-F1) stage liver fibrosis in treatment naïve HCV patients with an area under the curve, AUC = 0.848. **(B)** A moderate positive correlation is shown between the serum choline to uric acid ratio and independent FibroScan results from ultrasound imaging of HCV patients at different stages of liver fibrosis. **(C)** ANCOVA (between-subject effects) with covariate adjustments confirms that serum 5-oxo-proline concentrations are elevated in HCV patients with early and late-stage liver fibrosis as compared to non-HCV controls (*p < 0.05). **(D)** A weak positive correlation is depicted for serum 5-oxo-proline concentrations and FibroTest scores from a blood liver protein panel that differentiate non-HCV controls from HCV patients with chronic hepatic inflammation.

Figure 5A depicts a receiver operating characteristic (ROC) curve for the ratio of serum choline to uric acid based on MSI-CE-MS data, which provided optimal discrimination between HCV liver fibrosis patient sub-groups (AUC = 0.848, *p* = 0.00766) not feasible by serum liver protein panels or FibroTest scores (Table 1). Moreover, Figure 5B confirms that there was a moderate positive correlation (*r* = 0.606, *p* = 0.0047, *n* = 20) between the serum choline to uric acid ratio and liver stiffness measurements from FibroScan test results (kPa) in HCV patients. Also, an ANCOVA (between-subject effects) with adjustment for covariates (age, sex and BMI) revealed that serum 5-oxo-proline concentrations measured only by MSI-CE-MS were consistently elevated (mean FC = 1.44, *F* = 4.29, *p* = 0.0240) in HCV patients with early and late-stage fibrosis as compared to non-HCV controls as shown in Figure 5C. In contrast, liver stiffness measurements based on FibroScan test results were not able to differentiate non-HCV controls from early-stage fibrosis (F0-F1) HCV patients (Table 1). Figure 5D highlights that there was a weak correlation between serum 5-oxo-proline concentrations and FibroTest score (*r* = 0.349, *p* = 0.0586, *n* = 30), which is derived from an age/sex-adjusted algorithm of five serum liver proteins.

## Discussion

### Cross-Platform Serum Metabolomics Comparison

CE-MS based methods have not been widely used within the metabolomics community due to longstanding concerns regarding migration time variability and long-term robustness. However, these technical obstacles can be overcome with implementation of standardized protocols in large-scale metabolomic studies (Harada et al., 2018; Shanmuganathan et al., 2021). These protocols have been recently implemented in international ring trials to demonstrate cross-laboratory comparability (Drouin et al., 2020). Also, CE-MS inter-method comparisons increasingly demonstrate reliable quantification of metabolite concentrations relative to validated assays used in a clinical setting (DiBattista et al., 2017; Wild et al., 2019; Azab et al., 2020). Yet, there have been few cross-platform metabolomic studies involving CE-MS in conjunction with other widely used instrumental methods, such as LC-MS and GC-MS (Büscher et al., 2009; Naz et al., 2013; Rojo et al., 2015). To the best our knowledge, this work represents the first direct comparison between CE-MS and NMR for characterization of the serum metabolome following ultrafiltration.

There was considerable metabolome overlap between MSI-CE-MS and ^1^H-NMR with 20 metabolites consistently measured in serum filtrate samples, including amino acids, carnitines, organic acids, and glucose (Figure 3). MAGMET uses automated spectral processing, phasing, water-removal, baseline correction, chemical shift referencing, peak picking, curve fitting and spectral deconvolution via a biofluid-specific reference library for targeted quantitative profiling of 47 serum metabolites (Ravanbakhsh et al., 2015); however, only 30 serum metabolites were reliably measured in most non-HCV controls and HCV patients. This was likely due to sub-optimal shimming contributing to higher-than-average spectral line widths (∼0.95 Hz) and lower signal-to-noise for detecting several lower abundance metabolites (*e.g.,* hypoxanthine, acetoacetate) and organic solvents (*e.g.,* methanol, acetone) within the spectral library. In contrast, MSI-CE-MS takes advantage of both a targeted and nontargeted metabolomics data workflow with serum filtrates analyzed under two separation/ionization conditions for cationic and anionic metabolites (Figure 1); this process also allows for the discovery of unknown metabolites lacking authentic standards or reference data available in the Human Metabolome Database (Wishart et al., 2018). In this study, 60 serum metabolites (including five unknowns) were measured in most serum filtrates by MSI-CE-MS with good technical precision (CV < 10%) comparable to NMR (Figure 2). The greater serum metabolome coverage for MSI-CE-MS is mainly attributed to its improved resolution and lower detection limits as compared to 1D ^1^H-NMR. Both methods had good mutual agreement for 20 metabolites measured in 34 serum samples from non-HCV and HCV patients with a mean bias of 9.5% (*n* = 660) and few outlier data (Figure 3). Better quantitative performance in MSI-CE-MS may be realized when using matching stable-isotope internal standards with multiple reaction monitoring (Saoi et al., 2020) to correct for potential ion suppression effects unlike discovery-based metabolomics using a single internal standard with full-scan data acquisition. Furthermore, concentration sensitivity with deeper metabolome coverage can be further enhanced when using sheathless or low-flow CE-MS interface designs (Zhang et al., 2017).

Similarly, NMR quantification using automated MAGMET processing was prone to spectral interferences and potential bias, which can be minimized with lower detection limits when using higher field magnets. Overall, multiplexed electrophoretic separations based on MSI-CE-MS (Figure 1) enable faster data acquisition than NMR (<8 min/sample [both ion modes] vs 12 min/sample) with a two-fold greater metabolome coverage and analogous reproducibility. Additionally, MSI-CE-MS requires far less sample volume than NMR (<5 μl [if required] serum vs 450 μl serum) that is optimal for analysis of volume-restricted biospecimens (Nori de Macedo et al., 2017), and single-cell analyses (Duncan et al., 2019). Expanded metabolome coverage can be further realized when using non-aqueous buffers systems in MSI-CE-MS for a diverse range of water-insoluble yet ionic lipids and fatty acids from serum ether extracts (Azab et al., 2020).

### Serum Metabolites Signatures of Liver Fibrosis in Chronic Hepatitis C Virus Patients

Chronic HCV viral infections are one of several causes of liver disease, including fibrosis, cirrhosis, and hepatocellular carcinoma. Although liver biopsies remain the gold standard for diagnosis and staging severity, there is increasing use of ultrasound-based transient elastography for monitoring liver fibrosis progression as it correlates well with the METAVIR scoring system. However, this non-invasive method for assessing liver stiffness suffers higher failure rates for obese patients with ascites, and improved diagnostic confidence is achieved for mild disease states when combined with serum protein biomarker panels (Wilder and Patel, 2014). In our study, recruited HCV patients at different stages of liver fibrosis were well-matched (age, sex, BMI, HCV genotype, treatment naïve), with FibroScan test results differentiating moderate to severe fibrosis (F2-F3) or cirrhosis (F4) from no fibrosis (F0) or mild fibrosis (F1) patient sub-groups (Table 1). There was no difference in liver stiffness measurements when comparing early-stage liver fibrosis (F0-F1) HCV patients to a younger/healthy non-HCV control unlike the FibroTest score primarily from elevated γ-glutamyl transferase and alpha-2-macroglobulin levels in circulation. Despite aberrant metabolic signatures of fibrosis reported in various liver diseases (Chang and Yang, 2019), few metabolomic studies have identified serum biomarkers to differentiate liver fibrosis progression caused by HCV infection (Sarfaraz et al., 2016).

In this work, we identified four serum metabolites that differentiate fibrosis progression in HCV patients, including choline, histidine, proline and 2-hydroxybutyric acid. Interestingly, choline and histidine from serum filtrates were elevated in severe fibrosis/cirrhosis (F2-F4) as compared to early-stage fibrosis (F0-F2) HCV patients as independently confirmed by both MSI-CE-MS and NMR methods (Table 2). Choline is an essential dietary nutrient required for the biosynthesis of phospholipids and donor in methylation reactions, which plays a critical role in maintaining liver function (Mehedint and Zeisel, 2013). Although choline deficient diets can contribute to fatty liver and hepatic fibrosis, previous NMR metabolomic studies have shown that elevated serum choline in HCV patients differentiates hepatocellular carcinoma from HCV controls without liver cancer (Wei et al., 2012). Yet, contradictory findings were reported by NMR with lower levels of serum choline in hepatocellular carcinoma as compared to patients with liver cirrhosis (Gao et al., 2009). In our case, chronic HCV patients with hepatocellular carcinoma were excluded from study recruitment. However, more advanced stages of liver fibrosis/cirrhosis may progress to cancer without effective anti-viral medications. Additionally, serum histidine was previously demonstrated by NMR to be elevated in HCV patients with increasing liver fibrosis and necroinflammation (Safaraz et al., 2016). Histidine is an essential amino acid that also functions as an important antioxidant and metal chelator, which has been shown to attenuate thioacetamide-induced liver fibrosis in rats (El-Batch et al., 2011). Independent replication by two orthogonal instrumental methods further supports our findings in a well-matched patient cohort despite a modest study power involving primarily older male participants. Overall, the serum choline to uric acid ratio was found to improve discrimination between different stages of liver fibrosis (Figure 5A) as compared to choline alone (AUC = 0.803, *p* = 0.0317). In our study, lower circulating levels of uric acid were associated with more advanced fibrosis in largely overweight male HCV patients (Figure 4A), although hyperuricemia has been associated with liver damage in patients with non-alcoholic fatty liver disease (Afzali et al., 2010; Zhou et al., 2016). Importantly, there was a positive correlation (*r* = 0.606, *p* = 0.0047) in the serum choline to uric acid ratio with FibroScan test results, which links aberrant metabolism in circulation to liver disease phenotype/pathology in HCV patients (Figure 5B). Further validation in a larger patient cohort is needed to reproduce our findings and better demonstrate its clinical utility when used in conjunction with ultrasound imaging techniques.

Serum 5-oxo-proline as measured by MSI-CE-MS was found to be elevated in both early and late-stage fibrosis HCV patients as compared to non-HCV controls (Figure 5C). Oxo-proline (or pyroglutamic acid) is an important yet infrequently measured amino acid intermediate within the glutathione cycle (Bachhawat and Yadav, 2018), which accumulates in circulation due to acquired 5-oxoprolinemia from hepatic oxidative insult and glutathione depletion (Gamarra et al., 2019). However, most studies to date have focused on a high anion gap metabolic acidosis from 5-oxoprolinemia following sepsis or acetaminophen toxicity (Liss et al., 2013) rather than liver fibrosis/inflammation from chronic HCV infection or non-alcoholic steatohepatitis (Saoi et al., 2020). Serum cystine concentrations were also elevated among HCV patients with liver fibrosis relative to non-HCV controls in this study indicative of elevated oxidative stress in liver diseases (Cichoz-Lach and Michalak, 2014). In fact, serum 5-oxo-proline and cystine were highly co-linear (*r* = 0.619, *p* = 9.38 × 10^−5^, *n* = 34), yet they were not measured by NMR preventing their independent replication. FibroScan test results did not differentiate early-stage fibrosis HCV patients (F0-F1) from non-HCV controls without fibrosis unlike specific serum liver proteins, or FibroTest scores (Table 1), which were weakly correlated with oxo-proline concentrations (Figure 5D). Elevated circulating concentrations of oxo-proline reflecting oxidative stress and impaired glutathione-dependent redox homeostasis offers a plausible biochemical mechanism associated with the onset and progression of liver fibrosis in HCV patients.

## Concluding Remarks

We conducted the first cross-platform serum metabolomics study to compare the performance of MSI-CE-MS and NMR methods using standardized protocols, which was also applied to identify putative biomarkers of liver fibrosis from chronic HCV infection. Both techniques offered similar reproducibility with good mutual agreement and few outliers when quantifying 20 serum metabolites using a single internal standard. A targeted NMR metabolomics approach was facilitated by use of an automated spectral processing and deconvolution software together with a serum-specific metabolite library; however, sub-optimal shimming contributed to line width broadening and lower sensitivity with potential bias for certain serum metabolites prone to spectral interference. On the other hand, multiplexed separations by MSI-CE-MS offer faster data acquisition speeds, much lower sample volume requirements and greater metabolome coverage. Four serum metabolites were elevated in HCV patients with more advanced liver fibrosis severity, with choline and histidine being replicated independently by both instrumental platforms. Overall, the choline to uric acid ratio was found to optimally differentiate between late (F2-F4) and early (F0-F1) stages of liver fibrosis, which was also correlated well with liver stiffness measurements by ultra-sound imaging. Similar to serum liver protein panels, oxo-proline concentrations were higher in HCV patients with liver fibrosis as compared to non-HCV controls reflecting elevated oxidative stress and glutathione depletion from chronic inflammation. Further validation of serum biomarker candidates in this pilot study is warranted in a larger cohort of HCV patients while evaluating their clinical utility as compared to FibroTest scores and FibroScan test results. Serum biomarkers of hepatic fibrosis offer a less invasive procedure to liver biopsies when monitoring disease progression and treatment interventions for HCV patients to prevent end-stage liver failure.

## Materials and Methods

### Chemical and Reagents

All metabolite standards and buffers were purchased from Sigma-Aldrich (St. Louis, MO, United States). All LC-MS grade solvents, including acetonitrile, isopropanol, methanol, and water were obtained from Caledon Laboratories Ltd (Georgetown, ON, Canada). Calibrant solutions for serum metabolites were prepared by serial dilution of stock solutions (50 mM) in LC-MS grade water and stored refrigerated (4°C). A NMR Metabolomics Analysis Kit with access codes to MAGMET software were supplied by The Metabolomics Innovation Centre (Edmonton, AB, Canada). The kit includes Amicon filters with a 3 kDa molecular weight cut-off (MWCO), microcentrifuge tubes, NMR buffer (250 mM potassium phosphate, pH 7.0, 5 mM 2,2-dimethyl-2-silapentane-5 sulfonate, DSS-d_6_, 5.84 mM 2-chloropyrimidine-5-carboxylic acid, CPCA, and D_2_O 54% *vol* in H_2_O) and a QC standard mixture (1.25 mM glycine, 1.25 mM alanine, 1.25 mM threonine and 1.25 mM aspartic acid).

### Study Population and Sample Collection

The study approval was obtained from the McMaster University Health Research Ethics Board (REB Project #0932) and all study participants (20 patients with chronic hepatitis C and 14 participants as healthy controls) provided signed informed consent for study enrolment according to the Declaration of Helsinki. Relevant clinical and demographic information was also collected, including sex, age, body mass index (BMI), HCV genotype, cardiovascular risk factors (*e.g.,* hypertension, diabetes, and dyslipidemia), medication history (*e.g.,* lipid lowering therapies, oral hypoglycemics, and insulin), and habitual alcohol intake. An attending physician recruited patients if they meet the inclusion criteria while a research nurse, independent of the attending physician, obtained consent. Peripheral blood samples were collected during clinic visits. Following blood clotting (45 min at 25°C), the isolated serum samples were immediately transferred to cryovials and stored at −80°C. Routine clinical tests were also collected using Li-heparin, K-EDTA, and plain collection vials for total bilirubin, alanine aminotransferase, γ-glutamyltransferase, alpha-2-macroglogulin, haptoglobin and apolipoprotein A1. The study included patients chronically infected with HCV from the 2F Digestive Diseases Clinic at McMaster University (Hamilton, ON). Study inclusion criteria included: 1) adult patients (≥18 years of age) and 2) treatment naïve, chronic HCV patients (genotype 1, positive anti-HCV antibodies and HCV RNA in serum). Exclusion criteria included: 1) chronic hepatitis A, B, D and E, 2) conditions that may alter the accuracy of serum biomarkers of fibrosis: extra-hepatic biliary obstruction; immunosuppression (*e.g.,* due to HIV, medications); pregnancy; and systemic inflammatory conditions (*e.g.,* sepsis, inflammatory bowel disease), 3) excessive alcohol consumption defined as ≥ 40 g/d for men and ≥20 g/d for women, 4) antiviral therapy for HCV within the previous 6 months, 5) patients with NAFLD as determined by echogenic liver on ultrasound, and 6) patients with hepatocellular carcinoma. Percutaneous liver biopsy was performed under local anesthesia with an ultrasound guidance via the right costal approach (McGill, 2001). Although liver biopsies are considered the “gold standard” for staging fibrosis, there has been a decline in its use (Myers et al., 2014). FibroScan test results were used to non-invasively assess liver fibrosis progression in non-HCV and HCV patients at the Liver Clinic at McMaster University, and liver fibrosis was graded using the METAVIR scoring system. Also, the FibroTest score was calculated from a panel of five serum liver protein measurements after adjustment for age and sex.

### Serum Filtrate Preparation Prior to Metabolomic Analyses

Prior to NMR and MSI-CE-MS analysis, frozen serum samples were slowly thawed on ice and a pooled quality control (QC) sample was prepared by taking 10 µl aliquots of serum from each participant in the study. All serum samples were processed according to the protocol provided by the TMIC NMR metabolomics kit (http://magmet.ca/spectra_collection). The serum pre-treatment protocol was as follows: 3 kDa MWCO filters (Amicon Ultra 0.5 ml Centrifugal Filter Unit, Millipore Sigma Inc.) were first rinsed with 500 µl of water and centrifuged for 15 min at 14,000 *g* to remove additives from the manufacturing process. The rinsing process was repeated five times after which the filters were air dried prior to serum processing. An aliquot of 450 µl of serum sample was then added to the pre-rinsed filter tube and centrifuged for 25 min at 14,000 *g*. The serum filtrate containing free circulating polar/ionic metabolites (*i.e.,* non-protein bound fraction) was then separately aliquoted for independent MSI-CE-MS and ^1^H-NMR analysis. In this case, removal of serum protein by ultrafiltration reduced spectral interferences for metabolite quantification in NMR, as well as deleterious capillary surface adsorption and ion source contamination in MSI-CE-MS. An aliquot of 50 µl was used for MSI-CE-MS analysis while 280 µl was required for NMR analysis. The serum filtrate (280 µl) for NMR analysis was then diluted 1.25-fold with 70 µl of the NMR kit buffer and the solution was vortexed for 30 s and transferred to a 5 mm NMR tube. The serum filtrate (50 µl) for MSI-CE-MS analysis was diluted 4-fold in deionized water containing several internal/recovery standards, including 4-fluoro-*l*-phenylalanine (F-Phe, 20 µM), 3-chloro-*l*-tyrosine (Cl-Tyr, 20 µM), 2-naphthalenesulfonic acid (NMS, 20 µM), and ^13^C_6_-*d*-glucose (^13^C-glucose, 2 mM). The solution was vortexed for 30 s and a 20 µl aliquot was transferred into a polypropylene vial prior to MSI-CE-MS analysis.

### Nuclear Magnetic Resonance Data Acquisition

Data was obtained on a Bruker Avance III 700 MHz NMR spectrometer (Bruker Biospin, Rheinstetten, Germany) equipped with a 5 mm QNP cryoprobe, operating at 700.17 MHz for ^1^H and controlled by TopSpin software (v.3.5 for Linux OS). Data was collected at room temperature, using a noesypr 1d pulse program with water suppression (Ravanbakhsh et al., 2015). The acquisition and mixing time were set to 4 s and 100 ms, respectively. Spectra was acquired with eight steady state scans with a field width of ≤80 Hz and O1P and spectral width were set to 4.69 and 12 ppm, respectively. Each sample was shimmed using a manual shimming protocol which included Z6 shimming along the *Z*-*X*-*Y* axes before the Z-X-Y-XZ-YZ axes to maintain a peak linewidth for DSS-d5 (<1 Hz) required for automated MAGMET spectral processing. Each sample required about 12 min to complete 128 scans.

### Nuclear Magnetic Resonance Data Spectral Profiling and Annotation

The raw NMR data was uploaded in a zip format (FID file) to the MAGMET webserver (http://magmet.ca/users/login) that is based on the automated ^1^H-NMR spectral processing of serum filtrate samples as described previously (Ravanbakhsh et al., 2015). Briefly, a standardized workflow is used to process NMR spectral files, including Fourier transform/phase correction, baseline correction, water suppression, spectral smoothing, chemical shift referencing, followed by spectral deconvolution and metabolite quantification from a library of 47 serum metabolites. The following parameters were selected: the biofluid was set to serum, spectrometer frequency was set to 700 MHz, chemical shift (CS) reference and CS concentration were set to 4,4-dimethyl-4-silapentane-1-sulfonic acid (DSS-d6) and 1,000 μM, respectively. The internal DSS-d6 standard was used to calculate the concentration of all metabolites detected in the serum filtrates by comparing the peak area of individual metabolites in the spectra with the known concentration of DSS-d6. Also, CPCA was used for optimal automated phase correction based on its stable chemical shift at 8.76 ppm. A list of identified metabolites with their absolute concentration was outputted as a table format from the webserver. Serum metabolites detected in more than 75% of serum samples analyzed in this study were included in the data matrix for statistical analysis and any missing values and/or non-detects were replaced with half of lowest detected value.

### Serum Metabolomics by Multisegment Injection-Capillary Electrophoresis-Mass Spectrometry

An Agilent 6230 time-of-flight mass spectrometer (TOF-MS) with a coaxial sheath liquid Jetstream electrospray ion source with heated nitrogen gas was equipped to an Agilent G7100A capillary electrophoresis (CE) unit and used for the analysis of polar/ionic metabolites under aqueous buffer conditions. An Agilent 1260 Infinity Isocratic pump, and a 1260 Infinity degasser were used to deliver the sheath liquid at a rate of 10 μl/min. Separations were performed on an uncoated open tubular fused-silica capillary with an internal diameter of 50 μm and outer diameter of 360 µm (Polymicro Technologies Inc., AZ, United States) with a total capillary length of 135 cm. About 7 mm of the polyimide coating was removed from both ends of the capillary using a capillary window maker (MicroSolv, Leland, NC, United States) to reduce sample carry-over and prevent polymer swelling and/or degradation of the outer polyimide coating. Purine (10 µl) and hexakis (2,2,3,3-tetrafluoropropoxy) phosphazine (HP-921, 10 µl) were added into 200 ml of sheath liquid (0.1% *vol* formic acid in 60:40 MeOH:H_2_O, and 50:50 MeOH: H_2_O for positive and negative ion mode, respectively) to allow for real-time mass correction while also monitoring for potential matrix-induced ion suppression effects during separations since constant mass signals were detected at *m/z* 121.0509 and 922.0098 for purine and HP-921, respectively. The instrument was operated in 2 GHz extended dynamic range under positive and negative ion modes that spanned a mass range of *m/z* 50–1700. The data acquisition rate was set to 500 ms/spectrum and both profile and centroid data was stored in a “*.d” file format. The electrospray ionization conditions were set to, 2000 V for the Vcap and nozzle voltage during separation while turned off during injection, nebulizer was turned off during injection but was set to 10 psi during separation, while the drying gas was delivered at 8 L/min at 300°C with a sheath gas flow of 3.5 L/min at 195°C. In addition, the MS voltage settings for the fragmentor, skimmer and Oct1 RF were set to 120, 65, and 750 V, respectively. Instrument control and data acquisition were performed using Agilent MassHunter Workstation LC/MS Data Acquisition Software (B.06.01). New capillaries were conditioned by flushing at high pressure (900 mbar) with methanol for 30 min, 1.0 M NaOH for 30 min, de-ionized water for 30 min, and background electrolyte (BGE) for 30 min. At the start of each day, the CE electrode and MS interface was wiped daily with isopropanol:water (50:50) to avoid salt build-up followed by mass calibration of TOF-MS instrument as preventative maintenance measures.

All serum filtrate samples were analyzed by MSI-CE-MS under two configurations prior to a 10 min capillary flush with BGE namely an acidic BGE under positive ion mode for cationic/zwitterionic metabolites (1 M formic acid with 15% *vol* acetonitrile, pH 1.8), and an alkaline BGE under negative ion mode for acidic metabolites (50 mM ammonium bicarbonate, pH 8.5 adjusted with ammonium hydroxide). Serial sample injections in MSI-CE-MS were performed by alternating a hydrodynamic injection for each serum filtrate (100 mbar for 5 s) followed by an electrokinetic injection of BGE (30 kV for 75 s) to initiate electrophoretic separation at the capillary inlet. This interrupted separation process was repeated for a total of 13 serum samples that were introduced in a randomized order within a single run by MSI-CE-MS to ensure no effective loss in separation performance (Saoi et al., 2019). An applied voltage of 30 kV at 25°C was used for all runs in both positive and negative modes while a pressure gradient of 2 mbar/min was applied during separation (total time of 45 min) to allow for faster elution of slower migrating metabolites. BGE and sheath liquid were degassed before use by sonication for 10 min. Data normalization for peak integration used 20 μM 4-chlorotyrosine (Cl-Tyr) and naphthalene monosulfonic acid (NMS) as internal standards for positive and negative ion mode, respectively with the exception of glucose (total hexose) that used ^13^C_6_-glucose as it co-migrates with the electroosmotic flow (Shanmuganathan et al., 2021). A recovery standard, 3-fluorophenylalanine (F-Phe) was added to all serum samples prior to ultrafiltration to monitor for technical precision using control charts. Each sample effectively required 4 min to analyze by MSI-CE-MS in each ion mode while including a pooled quality control (QC) sample in every run. Metabolite identification for serum metabolites was confirmed (*e.g.,* co-migration, accurate mass) by spiking authentic standards in pooled serum filtrates. Serum metabolite quantification by MSI-CE-MS was achieved using a six-point calibration curve (in duplicate) over a 100-fold dynamic range with good linearity (*R* ^*2*^ ∼ 0.998) after least-squares linear regression with detection (S/N ∼ 3) and quantification (S/N ∼ 10) limits ranging from 0.2 to 0.5 μM to 1 and 2 μM, respectively as summarized in the excel file of the Supplementary Material.

### Statistical Analysis

Raw MSI-CE-MS data (*.d format) was processed using Mass-Hunter Workstation Qualitative Analysis software (version B.06.00, Agilent Technologies, 2012). A comprehensive study of all detectable molecular features from the raw data was performed using Mass-Hunter Molecular Feature Extractor, Molecular Formula Generator tools, and an in-house compound database. Molecular features were extracted using a symmetric 10 ppm mass window and all ions were annotated using their accurate mass (*m/z*), relative migration time (RMT) normalized to an internal standard (Cl-Tyr, NMS, or ^13^C-glucose), and ionization mode of detection (p: positive, n: negative). RMTs are reported since they are an important parameter used to exclude redundant adducts and/or fragment ion peaks, which exhibit identical RMTs as the parent compound. Peak smoothing was performed using a quadratic/cubic Savitzky-Golay function (7 points) prior to peak integration. Peak areas and migration times for all molecular features and internal standards were transferred to an Excel worksheet (Microsoft Office) and relative peak areas (RPA) for each unique molecular feature was saved as csv file. Molecular features detected in more than 75% of all serum samples analyzed with a coefficient of variance (CV < 35%) for QC samples were included in the final data matrix for further statistical analysis. Any non-detects were replaced by a value that was half the detection limit, where the limit of detection was set to the smallest value in the data set. Multivariate data analysis such as principal component analysis (PCA), partial least squares-discriminant analysis (PLS-DA) and receiver operating characteristic (ROC) curves were performed using the online webserver, MetaboAnalyst 5.0 (Chong et al., 2018), where data sets were (generalized) *log-*transformed (glog) and auto-scaled (PCA, PLS-DA) unless otherwise stated. Univariate statistical analysis, including student’s t-test and ANCOVA (between-subjects with adjustments for age, sex and BMI), and data normality test (Shapiro-Wilk test, α = 0.05) were performed using the Statistical Package for Social Science (IBM SPSS Statistical for Windows, Version 20.0. NY, United States). The inter-method comparison between MSI-CE-MS and ^1^H-NMR serum metabolite concentrations was performed using Bland-Altman %difference plots with MedCalc statistical software (MedCal^®^ Version 12.5, Ostend, Belgium). All electropherograms, mass spectra and graphs were displayed using Igor Pro (Wavemetrics Inc, OR, United States) or Microsoft Excel (Redmond, WA, United States).

## Data Availability

The raw data supporting the conclusions of this article are available in a public repository, MetaboLights (https://www.ebi.ac.uk/metabolights/).

## Abbreviations

m/z:RMT, accurate mass:relative migration time; BGE, background electrolyte; Cl-Tyr, 4-chlorotyrosine; CV, coefficient of variance; DSS, 2,2-dimethyl-2-silapentane-5-sulfonate; F-Phe, 3-flurorophenylalanine; HCV, hepatitis C virus; IS, internal standard; LC, liquid chromatography; MS, mass spectrometry; MAGMET, magnetic resonance for metabolomics; METAVIR, meta-analysis of histological data in viral hepatitis; MSI-CE-MS, multisegment injection-capillary electrophoresis-mass spectrometry; NMR, nuclear magnetic resonance; NMS, naphthalene monosulfonic acid; PCA, principal component analysis; PLS-DA, partial least squares-discriminant analysis; ROC, receiver operating characteristic curve.
